# Supplementary figures and images for: The winner takes it all: a single genotype of Kalanchoe × houghtonii is a global invader
Source: Ann Bot. 2025 Apr 24;136(1):179–98. doi: 10.1093/aob/mcaf076 (PMC12401883; doi:10.1093/aob/mcaf076)

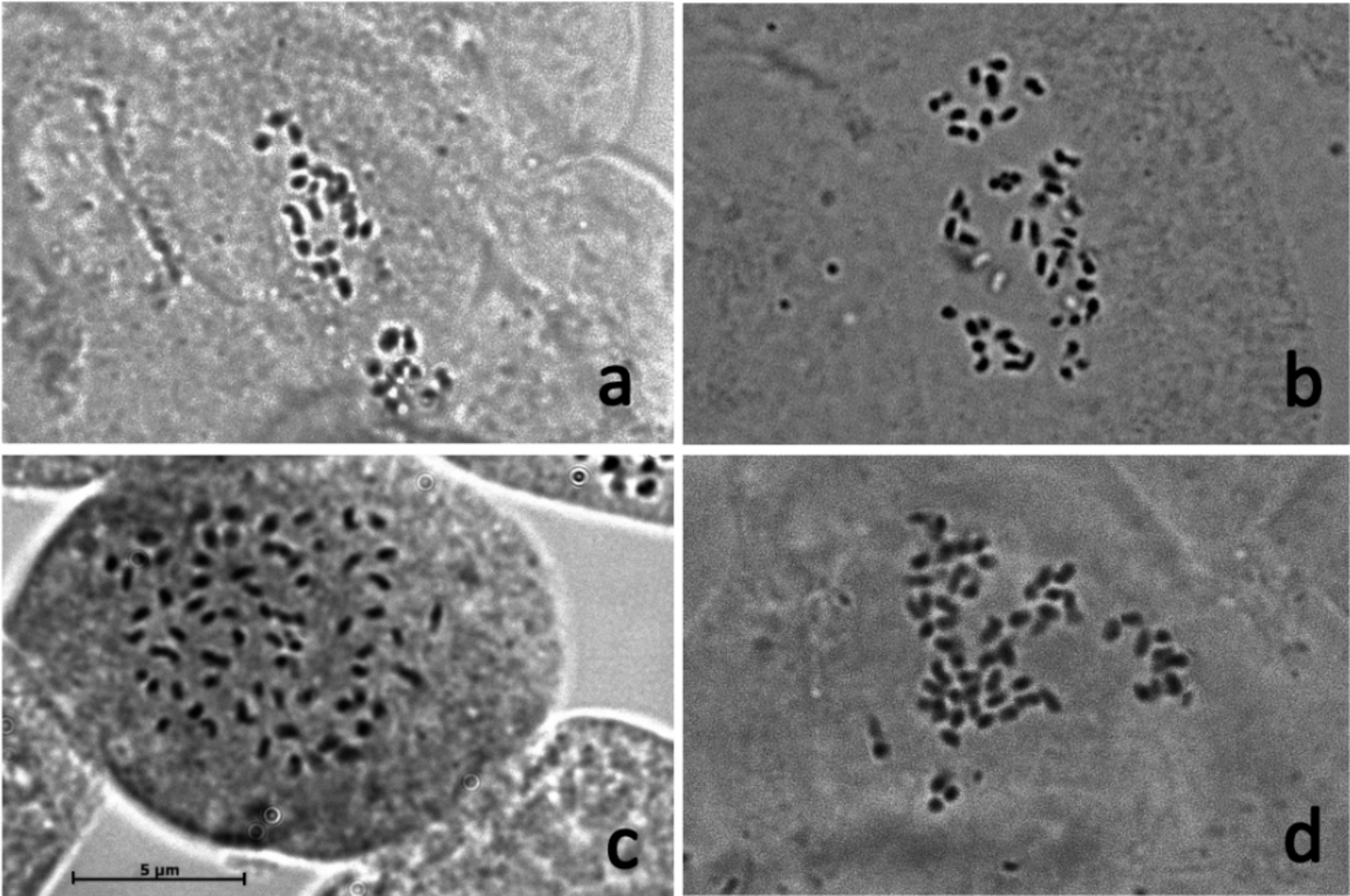

Supplement: mcaf076_suppl_Supplementary_Figures [file mcaf076_suppl_supplementary_figures.zip › mcaf076_suppl_Supplementary_Figures_S3.jpg]
